# The history of falls and the association of the timed up and go test to falls and near-falls in older adults with hip osteoarthritis

**DOI:** 10.1186/1471-2318-7-17

**Published:** 2007-07-04

**Authors:** Catherine M Arnold, Robert A Faulkner

**Affiliations:** 1 College of Kinesiology and School of Physical Therapy, University of Saskatchewan, Saskatoon, Canada; 2 College of Kinesiology, University of Saskatchewan, Saskatoon, Canada

## Abstract

**Background:**

Falling accounts for a significant number of hospital and long-term care admissions in older adults. Many adults with the combination of advancing age and functional decline associated with lower extremity osteoarthritis (OA), are at an even greater risk. The purpose of this study was to describe fall and near-fall history, location, circumstances and injuries from falls in a community-dwelling population of adults over aged 65 with hip OA and to determine the ability of the timed up and go test (TUG) to classify fallers and near-fallers.

**Method:**

A retrospective observational study of 106 older men and women with hip pain for six months or longer, meeting a clinical criteria for the presence of hip OA at one or both hips. An interview for fall and near-fall history and administration of the TUG were administered on one occasion.

**Results:**

Forty-five percent of the sample had at least one fall in the past year, seventy-seven percent reported occasional or frequent near-falls. The majority of falls occurred during ambulation and ascending or descending steps. Forty percent experienced an injury from the fall. The TUG was not associated with history of falls, but was associated with near-falls. Higher TUG scores occurred for those who were older, less mobile, and with greater number of co-morbidities.

**Conclusion:**

A high percentage of older adults with hip OA experience falls and near-falls which may be attributed to gait impairments related to hip OA. The TUG could be a useful screening instrument to predict those who have frequent near-falls, and thus might be useful in predicting risk of future falls in this population.

## Background

One out of three adults over the age of 65 and one out of two over the age of 80 falls annually[1]. Falling accounts for 77% of all elderly injury-related hospital admissions and is the cause of 57% of injury related deaths among elderly females and 36% among males in Canada[2,3]. In a review of 16 fall risk studies [1], presence of arthritis was identified as having a higher mean relative risk of predicting future falls than age or cognitive status; however, few studies have identified the type, location and related impairments and disabilities that might increase the risk of falls in this population. As well, there are no studies describing incidence of falls, near falls, or the type and circumstances of falls in individuals with lower extremity arthritis. Lower limb weakness, slower gait, decreased mobility and pain, all outcomes of hip OA, are also established risk factors for falls [4-6]. There is some evidence of increased fall risk in older adults with hip and knee arthritis [7]. However, others[8] found decreased fall risk for women with more severe radiographic changes of hip OA, but an increased risk for those with self-reported OA. This apparent paradox suggests that that those with more severe disease, due to more limited functional ability, may not put themselves at as great a risk compared to those with milder OA. Results from another study, showing patients with new episodes of hip pain had increased occurrence of falls, supports the notion of increased fall risk for those with milder or early signs of hip OA [9].

The timed up and go test (TUG) as described by Podsiadlo & Richardson[10] is a simple timed test to quantify functional mobility. The test requires the subject to stand up from a chair, walk 3 meters and return to a sitting position The TUG test has been associated with other tests of balance and functional mobility [10,11]. Recent studies support the predictive ability of the TUG to screen for older adults at risk for future falls[12,13], although others debate the sensitivity of this instrument to classify fallers[14] and there remains no clear cut-off score to predict high risk fallers[15,16]. The TUG has been found to be sensitive to functional change in patients following a total hip replacement[17] and as a predictor of falls six months following hip fracture surgery[13]; but, there are no studies evaluating the association of the TUG to fall risk in older adults with hip OA.

OA is one of the leading causes of disability in the elderly and by 2020 it is projected that the number of persons with arthritis will increase by 57% due to the expected increased number of older adults [18]. In Canada, long-term disability due to OA accounted for almost 80% of the nearly 3.5 billion total economic costs of arthritis in 1998 [19]. Identifying the number of falls, the nature, circumstances and injuries resulting from falls is important in designing fall prevention programs for this population. The primary purpose of this study was to describe fall risk, history and nature of falls and near-falls in community living adults over age 65 with hip OA. There has been little research on the history of near-falls in the community dwelling elderly although some suggest it is an important predictor of future falls[20]. These descriptive data are important in order to develop intervention strategies to reduce fall risk and fall incidence in a population that may be at higher risk than the healthy community dwelling elderly. A secondary purpose was to determine the association of the TUG test outcomes to fall and near-fall history in this population.

## Methods

### Participants and eligibility criteria

This study sample was also recruited for an exercise intervention study. Participants were recruited by newspaper advertisements and posters displayed in clinics, recreational facilities, senior residences and physician offices. Interested participants were first screened by a telephone interview in order to determine eligibility criteria and basic demographic information. The *telephone screening *included questions on age, presence and duration of hip pain, participation in various types of activities including frequency and duration, presence of other medical conditions, mobility rating, use of walking aid, and the frequency of falls in the past year. Activity level was categorized as: 1) limited (perform activities of daily living, but not involved in regular exercise, minimal walking outdoors), 2) light (gets outside walking or involved in light activities at least twice per week, duration less than 30 minutes) and 3) moderate (involved in moderate activity at least 2/week for 30 minutes or longer). Self-perceived mobility was rated on a scale of 1 to 10: 1 was defined as being dependent in a wheelchair and 10 as having no mobility problems. Co-morbidities identified were added for a cumulative score. Exclusion criteria included individuals: 1) with a medical or neurological disorder that significantly affected day to day function, 2) currently involved in a regular group exercise program 2 times per week or greater that incorporated aquatic exercise or balance activities (*criteria for the intervention study*), 3) reporting pain in the hip for less than 6 months or having no hip pain present or 4) who had joint replacement surgery within the last 6 months.

If participants were eligible based on the telephone screen, they were asked to attend a *physical screening exam *conducted by a physical therapist which included: 1) an interview confirming the frequency of falls and near falls within the past year, including details regarding the nature, circumstance and injury related to each fall recalled, 2) the Mini-Mental State Exam[21], 3) verification of presence of hip pathology using a clinical criteria, and 4) assessment of fall risk using the TUG test[10]. Prior to this screening test, participant consent was obtained. The study was approved by the institution's biomedical ethics review board.

### Falls and near-falls interview

A fall was defined as any event in which a person inadvertently or unintentionally comes to rest on the ground or another lower level such as a chair, toilet, or bed[22] A near-fall was defined as a slip (sliding of the support leg), trip (impact of the swinging leg with an external object) or loss of balance where the person starts to fall but is able to stop or prevent the fall to the ground or other lower surface[23] Participants were asked if they had a fall, and if so to recall the number of falls in the past year. Participants were also asked to describe where the fall occurred (indoors at home, outside at home, indoors in the community or outdoors in the community), the cause and circumstances related to the fall and if any injuries were sustained. Frequency of near-falls were categorized as frequent (occurring at least once per week or more), occasional (occurring less than 1/week but more than a couple times in the past year) or never. There are no data indicating the accuracy of reporting near-falls. Recognizing that near-falls are more difficult to recall than actual falls, this categorization criteria was thought to be more accurate by estimating the frequency of near-falls rather than re-calling specific events.

### Mini mental state exam

The mini mental state exam is a reliable interviewer-administered test of 11 questions to screen for cognitive impairment [21]. It was used in this study to identify participants who may have more difficulty recalling fall-related events and other demographic information. The maximum score on this test is 30 and scores of 20 or less have been found only in adults with a diagnosis of cognitive dysfunction[21].

### Determination of hip OA

**Figure 1:**
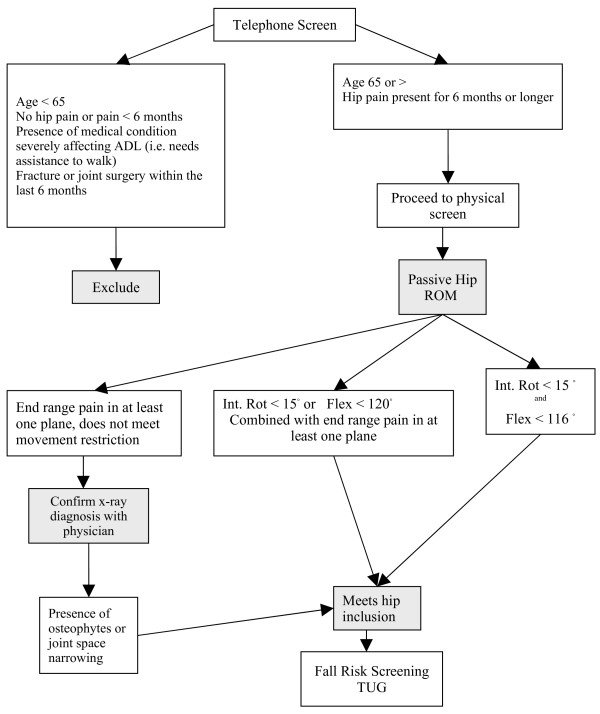
Screening criteria for inclusion in the study and classification of hip OA.

The classification system used to confirm hip pathology was based on the American College of Rheumatology (ACR) criteria to classify clinical presence of hip OA when radiographs are not available (Figure 1). As per the ACR recommendations, the measurement of both internal rotation and flexion of the hip was used with flexion restriction as a secondary criteria for determining presence of joint disease (87% and specificity of 75% [24,25]). Where pain on hip motion was present, but movement restriction did not meet the criteria, reports from the most recent hip radiograph were used to confirm diagnosis. Health professionals are often in the situation needing to distinguish the presence of pain associated with joint disease as opposed to radiating pain in the hip region from the spine, bursa, or surrounding musculature. The presence of end range pain in internal rotation and flexion with a secondary restriction of abduction is a commonly reported capsular pattern of restriction for the hip joint [26]. The presence of hip pain for at least 6 months, also rules out short-term pain conditions.

### TUG

The TUG test was used as a test for functional mobility and fall risk [10]. A standard chair with armrests was used for all tests. The participant was asked to stand up, using the armrests as necessary, walk past a line 3 meters away, turn around and come back and sit down in the chair. Participants were timed from the point where their buttocks rose from the chair to when their buttocks touched the chair when returning to sitting. The instructions were to walk (not run) as quickly, but as safely as possible. [15] Participants had one practice trial, and the second trial was timed. If a walking aid was usually used inside the home, then the walking aid was used during the test. This test has been found to be reliable and sensitive to functional change in the older adult population with hip dysfunction(ICC = 0.75 – 0.99 [10,13,17]).

## Analysis

The description of all fall events were categorized for mechanism or cause of fall, the activity the faller was doing at the time of the fall, the environmental location of the fall and any injuries that were incurred. Responses were reviewed by the researcher and categorized into common themes based on previous literature[27,28]. Injuries from falls were categorized as: 1) fracture, 2) other soft tissue or joint injury (not including simple abrasions or cuts, and 3) no injury. Seeking medical treatment or emergency room care for an injury was not used to categorize injury as it was felt that many fall-related injuries may not be reported to a medical practitioner.

Descriptive statistics and frequency data were generated for demographic information and the TUG scores. Descriptive statistics were calculated for the prevalence of fall and near-falls, and compared among three TUG score categories: < 10 seconds, 10 – 13.99 sec and 14 sec or >. These categories represented the lowest and highest quartiles found in this sample and were similar to other cut-off TUG scores reported in previous literature[11,29]. A one-way analysis of variance was used to compare age, medication use, mobility rating and number of co-morbidities among the three TUG categories. Odds ratios were calculated to examine the association of the TUG test to fall and near-fall history. Odds ratios for being a faller vs. non-faller or a frequent near-faller vs. occasional or never were calculated for TUG categories of < 10 seconds compared to > 10 seconds and less than 14 seconds compared to > 14 seconds, the lowest and highest quartiles. Odds ratios were calculated for other factors converted to dichotomous variables (activity level, age, location of hip pain and use of a walking aid). Receiver operator characteristic (ROC) curves were generated for the association of TUG scores to fall history and TUG scores to history of frequent near-falls with sensitivity on the y-axis and 1 – specificity on the x-axis. The area under the ROC curve reflects the degree of accuracy of the TUG in classifying fallers and frequent near-fallers. A value of 1.0 is an ideal test with 100% sensitivity and 100% specificity. A value of 0.5 represents 50% sensitivity and 50% specificity, a test with no discriminative value. Screening characteristics were determined for all cut-offs between 10 sec and 14 sec of the TUG.

## Results

### Participants

**Figure 2:**
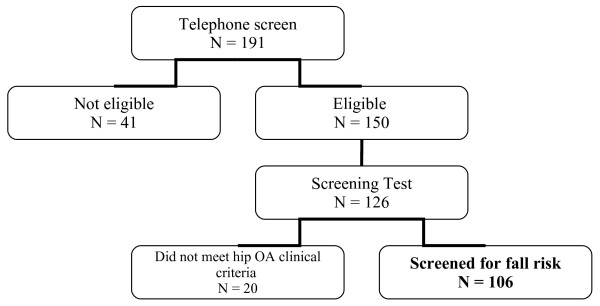
Flow chart of participants included in the study.

One hundred and ninety-one participants were telephoned screened and 41 of these were excluded (only 5 as a result of being too active for the intervention study). One hundred and twenty-six of these participants agreed to attend a physical screen. The mean score on the Mini Mental State Exam was 28.2 (2.0) out of a possible score of 30. All participants scored 22 or greater on the Mini Mental State Exam, and only one participant scored less than 24. Twenty did not meet hip OA eligibility, resulting in a final sample of 106 eligible participants for the study (Figure 2). One hundred and five participants completed the TUG as one participant was assessed as not safe to complete the test. Of the 106 screened, 77 were female (73%) and 29 were male (27%). Fifteen participants (14%) had a previous total or partial hip arthroplasty more than 6 months ago. Seven individuals were on a waiting list for a total hip arthroplasty. Other descriptive data of the sample are reported in Tables 1 and 2.

**Table 1:** Ambulatory and clinical characteristics of participants (n = 106)

Variable	Frequency	Percent
Exercise level		
• Limited	42	39.6
• Light	44	41.5
• Moderate	20	18.9

Concurrent conditions		
• Osteoporosis	41	38.7
• Knee OA	21	19.8
• Arthritis in other joints	35	33.0

Use of walking aid	40	37.7
Type of walking aid		
• 1 cane	25	62.5
• Walker	7	17.5
• 2 canes	3	7.5
• Both walker and cane	5	12.5
Use of walking aid		
• Outdoors only	24	60.0
• Both in and outdoors	16	40.0
Lives alone*	29	39.7
Previous fracture**	28	33.7

Hip affected		
• Right	40	37.7
• Left	25	23.6
• Both	41	38.7

* n = 73, ** n = 83

**Table 2:** Descriptive data for demographic and TUG scores

**Variable**	**Mean (SD)**	**Range**	**N**
Age	74.4 (6.2)	65 – 88	106
Total co-morbidities	2.2 (1.3)	0 – 7	106
Total prescription medications	3.0 (2.6)	0 – 12	81
Total non-prescription medications	2.5 (2.0)	0 – 8	81
Length of time with OA	8.1 (8.3)	0 – 50	73
Mobility rating	6.5 (1.8)	1 – 10	101
TUG score	12.8 (5.3)	6.2 – 37.5	105

### Falls and near-falls

The frequency of falls and near falls, fall mechanism, location and any injuries sustained is reported in Table 3. Forty-five % of the sample had at least one fall in the past year. A total of 59 falls were recalled from 48 respondents. A secondary analysis was done comparing frequency of falls in two age groups. Forty percent of the group under the age of 75 fell in the past year and 52% of those aged 75 and older fell in the past year. Trips were the primary cause of falls followed by slips and lost balance. Lost balance included a broad range of responses such as falling for no apparent reason, or generally losing balance when standing on an unstable surface or in a static position. Ambulation (not on stairs or over curbs) was the most common activity where falls occurred, followed by ascending or descending stairs and reaching and getting up from a chair or bed. In the reaching category, 4 falls occurred when standing on a chair, ladder or step and reaching for an object. Ten percent of falls reported resulted in a fracture. The fractures that occurred included 1 distal radius, 1 spinal compression, 1 rib, 1 hip and 2 clavicle or shoulder girdle. Almost 80% of the sample reported frequent or occasional near-falls where they lost their balance, but they were able to recover before landing on the floor or other lower surface.

**Table 3:** Frequency of fall, near-falls, mechanism, location and injuries sustained from falls

**Variable**	**Frequency**	**Percent**
Participants reporting at least 1 fall in past year		
	48	45.3

Frequency of falls		
• 1 fall	37	77.1
• 2 or more falls	11	22.9

Location of fall*		
• In home or residence	29	49.2
• Outside the home or residence	10	17.0
• Indoors in the community	5	8.5
• Outdoors in the community	15	25.4

Mechanisms or causes of the fall*		
• Tripped (impact of swing leg on external object)	21	35.6
• Slipped (sliding of support leg)	16	27.1
• Lost balance	15	25.4
• Missed curb or step	4	6.8
• Muscle weakness/leg gave away	3	5.0

Activity at time of the fall		
• Ambulating	33	55.9
• Ascending or descending stairs or step	13	22.0
• Reaching	7	11.9
• Getting up or down from chair or bed	6	10.2

Injuries sustained from falls reported*		
• Fracture	6	10.2
• No fracture, but other injuries beyond minor scratch or bruise	18	30.5
• No injury	35	59.3

Frequency of Near falls**		
• Frequent (1/week or more)	31	29.8
• Occasional (< 1/week but more than once or twice in past year)	49	47.1
• Never	24	23.1

* Total of 59 falls recalled by 48 fallers; ** n = 104

### Association of TUG scores to fall risk

As shown in Table 4, there were significant differences (p < 0.05) for age, number of co-morbidities, number of prescription medications, and mobility rating, when comparing the highest quartile of the TUG scores (14 sec. or >) with the lowest (< 10 sec.). Age and mobility rating were significantly different between the mid range (10 – 13.99 sec) and the lowest quartile (< 10 sec.).

**Table 4:** Mean values and standard deviations for age, mobility and other demographic factors comparing three TUG categories: < 10 seconds, 10 – 13.99 sec. and 14 or > sec.

Variable	< 10 sec.	10 – 13.99 sec.	14 or > sec.
Age (n = 106)	70.4 (4.2)	73.5 (5.5) *	79.7 (5.7) * ^†^
Mobility rating (1–10; n = 100)	7.4 (1.8)	6.4 (1.7) *	5.5 (1.4) *
# prescription meds (n = 81)	1.5 (1.9)	2.8 (2.8)	4.0 (2.0) *
# co-morbidities (n = 105)	1.6 (0.9)	2.1 (1.1)	2.9 (1.6) * ^†^
Length of time hip OA (yrs; n = 73)	5.8 (5.6)	7.6 (7.2)	10.4 (10.9)

* p < 0.05 comparing to < 10 sec.; ^†^p < 0.05 comparing to 10 – 13.99 sec. category using Tukey's post-hoc analysis

**Figure 3:**
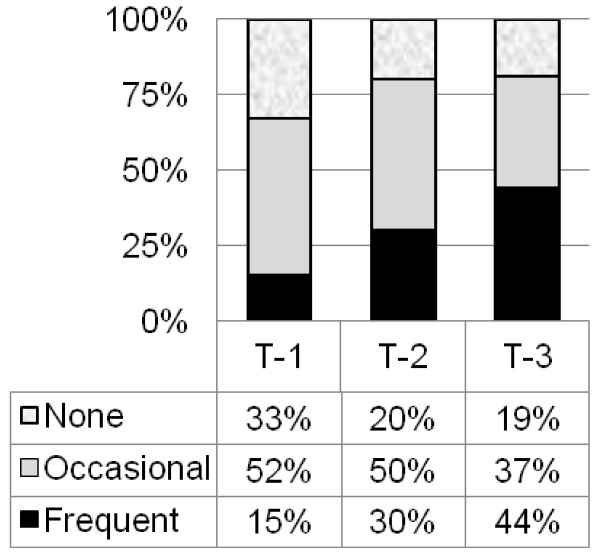
The frequency of near-fallers for three TUG categories: < 10 seconds, 10 – 13.99 seconds and 14 seconds or >. Key for Figure 3: T-1: TUG score < 10 seconds. T-2: TUG score 10 seconds – 13.99 seconds. T-3: TUG score 14 seconds or >.

There were no significant differences in the number of fallers and the frequency of near-falls among the three TUG categories; but the distribution for near-fall frequency was closer to what was expected with a trend of increasing percentage of frequent near-fallers in the higher TUG scores and declining numbers of participants with no history of near-falls (Figure 3). Based on calculation of odds ratios (Table 5), participants were three times more likely to be a frequent near-faller if their TUG score was > 10 seconds or if they were over the age of 75. The odds ratio did not increase substantially using a higher cut-off for the TUG of 14 seconds for the association to a history of falls or near-falls. Because age was a potential confounder in determining the relationship of the TUG to fall and near-fall history, a post-hoc analysis was done comparing odds ratios in two groups: under age 75 and 75 years or older. The odds ratios associated with being a near-faller remained similar for both age groups: OR = 3.0 (CI 0.44 – 20.4) and OR = 2.5 (CI 0.24 – 25.7) for the younger and older group respectively. The association of TUG scores to fall history remained low and inconsistent for the two age subgroups, with no association found for TUG scores to fall history in either group. There were no other significant associations found for the other dependent variables (gender, use of walking aid, mobility level or hip pain bilateral vs. unilateral) to frequent near-falls, and there were no significant associations of TUG scores or any other factor to fall history (Table 5).

**Table 5:** Odds ratios and 95% confidence intervals for predicting fallers and frequent near-fallers

	Faller OR (95% CI)	Frequent near-faller OR (95% CI)
Uses walking aid vs. none	0.84 (0.4 – 1.8)	1.4 (0.6 – 3.3)
Age 75 + vs. < 75	1.6 (0.8 – 3.6)	**3.0 (1.3 – 7.3)**
Hip pain bilateral vs. unilateral	0.9 (0.4 – 2.2)	0.5 (0.2 – 1.4)
Limited activity vs. light or moderate	0.9 (0.4 – 1.9)	2.2 (0.9 – 5.1)
Female vs. male	1.0 (0.4 – 2.3)	0.7 (0.3 – 1.9)
TUG score 10 sec or > vs. < 10 sec	1.0 (0.4 – 2.3)	**3.1 (1.0 – 9.9)**
TUG score 14 sec. or > vs. < 14 sec.	1.4 (0.6 – 3.4)	2.4 (1.0 – 6.1)

Further analysis of ROC curves showed that the area under the curve for TUG score and fallers was 0.58 (95% CI 0.47 – 0.70). The area under the curve for frequent near-fallers for the TUG was 0.65 (95% CI 0.53 – 0.76). Thus, TUG was a more accurate test in predicting frequent near-fallers than fallers, but with an area under the curve of 0.65 it did not demonstrate a high level of accuracy. Sensitivity and specificity values for TUG categories from 10 sec. to 14 sec to classify fallers and frequent near-fallers are reported in Table 6. The cut-off of 10 seconds showed the highest degree of sensitivity for both fallers (73%) and frequent near-fallers (81%). The sensitivity or the ability to detect those at risk is important in falls screening. There was a forty-five percent increase in sensitivity for the 10 second cut-off as compared to the cut-off of 14 sec. A cut-off of 11 sec. improved specificity by 20% with only a small drop in sensitivity of 6% for classifying fallers; however this was not true for classifying frequent near-fallers as there was an equally high drop in sensitivity as specificity from a cut-off of 10 seconds to 11 seconds.

**Table 6:** Test Characteristics of TUG for identifying fallers and frequent near-fallers

**TUG cut-off score**	**10 sec**	**11 sec**	**12 sec**	**13 sec**	**14 sec**
N (TUG ≥)	77	56	38	30	27
Sensitivity Faller	0.73	0.67	0.44	0.33	0.27
Specificity Faller	0.35	0.55	0.70	0.74	0.77
Sensitivity Near-faller	0.81	0.68	0.55	0.45	0.36
Specificity Near-faller	0.36	0.51	0.70	0.77	0.79

## Discussion

The first purpose of this study was to describe the one year history and circumstances of falls in a population of older adults with hip pain due to osteoarthritis. The nature of falls and near-falls for individuals with hip OA has not been previously described despite evidence that the presence of lower extremity arthritis significantly increases fall risk[7].

We found that 45% or approximately 1 out of 2 older adults with hip OA fell at least once annually. This is higher than the commonly estimated prevalence of 30% or one out of 3 older adults over the age of 65 living in the community falling annually [1,30]. Although there was a higher percentage of fallers aged 75 or older, there were 40% who experienced a fall in the past year under the age of 75. All participants were independently living in the community with no cognitive impairments; therefore the higher prevalence of fall history likely was not simply a factor of sample demographics. As well, 80% reported more than one occasion of a near-fall in the past year and 30% reported that near-falls occurred at least once per week.

Few studies have attempted to identify the number of near-falls reported by the elderly. Ryan et al in a small sample of community living elderly found that 70% reported a near-fall in the past month[23]. Using the definition of a "stumble" as loss of balance regained before landing on the ground or another object, Teno et al (1990)[20] found that adults who reported two or more stumbles were twice as likely to experience a subsequent fall. One of the reasons for the lack of further data on near-fall events is the difficulty of recall. We attempted to avoid the difficulty of recall by asking participants to estimate frequency of near-falls, rather than specific events. Other screening tools have utilized this approach[31]. Although it is not clear if this is a more accurate method of estimating near-falls rather than recalling specific events, it does provide an estimation of how often older adults lose their balance in daily activities, something not previously reported in the literature. Cummings et al[32] found that older adults were more likely to under-report fall events (forget a fall that occurred) verses over-reporting (recalling a fall that had not occurred). As well, individuals who scored higher on MMSE were more likely to be accurate in their fall related history[32]. In this study, there were no signs of cognitive impairment based on scores of the MMSE or during the interview procedure. As well, Hale et al reported a high level of accuracy (92%) in older adults recalling fall related events in the past year[33]. Although one could argue that recalling the frequency of near-fall events may be more difficult than an actual fall, 30% reported near-falls occurring as recent as one week or less.

The relatively higher frequency of falls and near-falls reported in this study may be a factor of the activities and circumstances related to the fall. For example, almost 80% reported falling during ambulation or while climbing or descending stairs. In contrast, other studies in older adults have found that approximately 50% of falls reported are related to ambulation activities[27,29] When walking, an individual must bear 80% of her/his body weight on a single limb for 60% of the gait cycle; this equates to a loading force through the hip greater than 4 times the body weight [34]. Individuals with hip pain and/or muscle weakness surrounding the hip will often compensate for the decreased ability to support load on one limb by shifting the center of body mass over the support limb in order to increase the efficiency of the abductor muscles [34]. This compensation results in an abnormal gait with a displacement of the center of gravity toward the side of the painful hip. As a result, balance may be jeopardized and risk of falling increased; the risk is particularly increased if combined with environmental obstacles, poor visual cues or decreased proprioception. This sequence of events is compounded further by age-related changes in gait: slower speed, decreased stride length, increased double support, decreased plantar flexion propulsion, and decreased hip extension[36,37]. There is evidence that loss of hip extension range and strength may be biomechanical contributors to fall risk[38,39].

Other activities reported where falls occurred included reaching and getting up from a chair or bed. These activities could also be directly associated with impairments related to having hip OA as it requires weight shifting on one lower extremity to reach and adequate strength in hip musculature to move from sitting to standing. It is interesting and somewhat alarming that 4 out of the 7 falls related to reaching for articles were due to standing on a chair, ladder or stepping from a chair to a ladder in order to reach a high object.

Tripping was the most common reason reported for falling, followed by slipping. Biomechanical studies suggest that the ability to prevent a fall in the event of a trip depends on where the center of gravity is located at the time of the trip. An anterior shift of the center of gravity due to flexed posturing or loss of hip extension is associated with falling when a trip is induced. Buckling of the limb, which can occur due to pain or muscle weakness, is also associated with a greater risk of falling[39,40]. The accumulation of gait adaptations due to hip pain from OA, combined with aging could increase the risk of falling during locomotion in this population. Other mechanisms for falls reported such as slipping, missing a step, leg giving away or just losing balance could also be associated with the presence of pain, loss of range and weakness due to hip OA.

Most falls occurred in the participant's own home or residence or just outside their residence. This result is consistent with other studies of older adults and highlights the observation that most falls occur in very familiar surroundings and are not due to an unexpected environmental hazard. Ten percent of our sample sustained a serious injury as the result of the fall (fracture reported). Most other studies report injury rates in the range of 1.5% [27] to 6%[41]. Although there has been some evidence suggesting the incidence of fragility fracture is lower in individuals with OA due to increased bone density in bone surrounding OA joints, others have found that the incidence of fragility fracture is not decreased in older adults with OA [8,42]. Our data suggest that the annual incidence of fragility fracture due to falls is just as high, if not higher than other findings in the community dwelling elderly. This greater incidence could be due to the increased exposure to trauma from a higher number of falls occurring, although the sample in this study is not large enough to make any definite conclusions.

The second purpose of this study was to determine the usefulness of the TUG test to classify fallers and frequent near-fallers in older adults with hip OA. The TUG test is a commonly used screening test for mobility dysfunction and as a predictor for fall risk in the elderly. Although the test has been recommended as a sensitive measure to predict future falls post hip fracture surgery[13], others have cautioned its usefulness in predicting fall risk. Similar to our results, Thrane et al[14] found the ability of TUG to classify fallers retrospectively was poor. We found that the TUG was not associated with a history of falls in men and women with hip OA, and its ability to classify fallers was poor. It appeared that the TUG was better at distinguishing mobility difficulties related to reports of frequent near-falls as opposed to fall history. The TUG had a stronger association to a history of frequent near-falls (once a week or more) than to actual falls in older adults with hip OA. This relationship held true for older adults whether they were aged 65 to 74 or aged 75 or older. If participants scored 10 seconds or > they were three times as likely to be a frequent near-faller. The highest sensitivity to predict frequent near-fallers was a cut-off of 10 seconds. This is lower than other TUG values recommended for predicting future fall risk such as 16 seconds[13] and 13.5 seconds[15]. However, Whitney et al[43] reported a cut-off score of 11.1 seconds resulting in sensitivity of 80% and specificity of 56% in classifying retrospective fall history. Because this was not a prospective study, conclusions about the best cut-off score to use for predicting future fall risk must be done with caution. Nevertheless, our results, also suggest that using a higher value for the TUG (i.e. 14 seconds) to predict falls may miss many older adults with a moderate to high risk of falling.

If near-falls are a good predictor of future falling, then the TUG could be a useful indicator for risk of future falls. Similar to other studies, we found that the profile of older adults that score less than 10 seconds on this test were healthy community living adult who were younger, taking fewer prescription medications and were more functionally independent [10,11]. However, a lower score on the TUG did not translate to fewer retrospective reports of falls. Therefore the TUG appears to have greater use for older adults with hip OA in its ability to predict mobility loss and frequency of near-fall events rather than its ability to classify fallers vs. non-fallers.

Results of this study provide important information on falls and near-falls in a population rarely studied (older adults with hip OA); however our data has several limitations. First, the participants who responded may not be representative of fall history of the general population with hip OA; that is, it's possible that participants self-selected because they were interested in reducing their fall risk. It was impossible to not inform participants of the intention of the study, and it is unlikely that there was a bias toward higher risk fallers volunteering. From the demographic data of age, number of co-morbidities, prescription medications and mobility level, this sample seemed very reflective of the community living older adult. One of the participant exclusion criteria included higher activity levels, as this study was the initial screening for an exercise intervention study. However, only 5 participants were excluded based on the physical activity exclusion criteria. In addition, the percentage of participants classified as moderately active was 19%. This compares favorably to Jerome et al, who reported that less than 15% of a sample of 710 women aged 70–79 with self-reported functional deficits participated in moderate activity for 150 minutes or more per week[44]. The 39% of this sample who reported zero minutes of moderate physical activity per week is also consistent with Canadian statistics for community dwelling elderly where 23% to 40% of older adults report limited activity [45]. Finally, the size of this sample was not sufficient to thoroughly test the ability of the TUG to classify fallers. Future study needs to do a prospective analysis of the ability of the TUG to predict falls in older adults with hip OA.

## Conclusion

In conclusion, one out of two adults aged 65 and older with hip OA fell at least once in the past year, more than reported in the healthy community living older adult population. Most of these falls occurred during ambulation and when navigating steps and stairs, which may reflect impairments in gait often associated with hip OA. The TUG test was not an effective discriminator of previous fallers and non-fallers. But, TUG scores were related to near-falls incidence; thus it may be a useful tool in screening older adults for mobility difficulties associated with balance and future fall risk.

## Competing interests

The author(s) declare that they have no competing interests.

## Authors' contributions

Both authors have made substantial contributions to all aspects of conception, design, data collection, analysis, interpretation and writing of the manuscript. Both CA and RF contributed to conception and design of the study. CA conducted data acquisition and data analysis, in consultation with RF. Both authors participated in interpretation of the data. CA drafted the manuscript with critical revisions by RF.

## Pre-publication history

The pre-publication history for this paper can be accessed here:


